# Mothers' amygdala response to positive or negative infant affect is modulated by personal relevance

**DOI:** 10.3389/fnins.2013.00176

**Published:** 2013-10-08

**Authors:** Lane Strathearn, Sohye Kim

**Affiliations:** ^1^Attachment and Neurodevelopment Laboratory, Department of Pediatrics, Children's Nutrition Research Center, Baylor College of Medicine, HoustonTX, USA; ^2^The Menninger Department of Psychiatry and Behavioral Sciences, Baylor College of Medicine, HoustonTX, USA; ^3^The Meyer Center for Developmental Pediatrics, Texas Children's Hospital/Baylor College of Medicine, HoustonTX, USA

**Keywords:** amygdala, valence, relevance, mother-infant, faces, functional MRI, emotion

## Abstract

Understanding, prioritizing and responding to infant affective cues is a key component of motherhood, with long-term implications for infant socio-emotional development. This important task includes identifying unique characteristics of one's own infant, as they relate to differences in affect valence—happy or sad—while monitoring one's own level of arousal. The amygdala has traditionally been understood to respond to affective valence; in the present study, we examined the potential effect of personal relevance on amygdala response, by testing whether mothers' amygdala response to happy and sad infant face cues would be modulated by infant identity. We used functional MRI to measure amygdala activation in 39 first-time mothers, while they viewed happy, neutral and sad infant faces of both their own and a matched unknown infant. Emotional arousal to each face was rated using the Self-Assessment Manikin Scales. Mixed-effects linear regression models were used to examine significant predictors of amygdala response. Overall, both arousal ratings and amygdala activation were greater when mothers viewed their own infant's face compared with unknown infant faces. Sad faces were rated as more arousing than happy faces, regardless of infant identity. However, within the amygdala, a highly significant interaction effect was noted between infant identity and valence. For own-infant faces, amygdala activation was greater for happy than sad faces, whereas the opposite trend was seen for unknown-infant faces. Our findings suggest that the amygdala response to positive or negative valenced cues is modulated by personal relevance. Positive facial expressions from one's own infant may play a particularly important role in eliciting maternal responses and strengthening the mother-infant bond.

## Introduction

Motherhood provides the earliest laboratory for infant social learning, and plays a critical role in shaping infant socio-emotional development (Sroufe, 2005; Feldman, 2007; Strathearn, 2011; Mills et al., 2013). Rodent models of maternal behavior have defined neurobiological mechanisms by which contingent, responsive maternal caregiving may promote social development and regulate stress across generations, at least partially via regulation of oxytocin and central benzodiazepine receptor expression in the amygdala (Caldji et al., 1998; Francis et al., 1999; Champagne et al., 2001).

While struggling to meet competing demands for time and attention, mothers must frequently appraise their infants' emotional cues and prioritize responses to the most salient of these cues. Happy or smiling infant face cues are particularly motivating for mothers, and have been shown using functional MRI (fMRI) to activate brain regions involved in reward processing (Strathearn et al., 2008) and attachment (Strathearn et al., 2009). They may also play an important role in promoting mother-infant bonding, by eliciting reciprocal smiles and playful interactions with caregivers, and thus enhancing socio-emotional development in infancy (Minagawa-Kawai et al., 2009; Bigelow et al., 2010).

Sad infant faces, often accompanied by a powerful auditory cue—infant cry—are also important signals relating to infant need, whether it be for food, rest, warmth or attention. For mothers, hearing infant cries activates a range of brain areas related to maternal caregiving behavior (Lorberbaum et al., 2002), with amygdala activation to cries also related to maternal sensitivity (Kim et al., 2011) and the development of infant attachment (Laurent and Ablow, 2012). While sad face cues elicit a *reactive* parental response, happy face cues tend to elicit a *proactive* response leading to positive social experience such as interactive play, physical touch, tickling, kissing and caressing.

So how do mothers interpret and prioritize their responses to infant affective cues—positive or negative? Is it, for example, more important to engage with their smiling infant or to respond to physical needs that may provoke a sad face or cry? How do maternal responses differ when engaging with one's own infant compared with someone else's infant? The amygdala is a key component of the brain's neural network that specializes in emotion processing (Murray, 2007), particularly as expressed in human faces (Costafreda et al., 2008; Sergerie et al., 2008; Atkinson and Adolphs, 2011). Originally characterized as the “fear center” of the brain, based on studies of fear conditioning (Rosen and Donley, 2006; Sehlmeyer et al., 2009), the amygdala was thought to function primarily as an alert system to protect oneself or significant others from potential threat. Several functional MRI studies have demonstrated amygdala activation in mothers viewing their own vs. other child face cues (Leibenluft et al., 2004; Ranote et al., 2004; Strathearn et al., 2008; Barrett et al., 2011), interpreted by some to indicate mother's “vigilant protectiveness” toward her own child (Leibenluft et al., 2004; Gobbini and Haxby, 2007). However, other studies have provided conflicting evidence, including one revealing amygdala *de-activation* (Bartels and Zeki, 2004), and others not finding any significant amygdala activation to own vs. unknown infant faces (Noriuchi et al., 2008; Lenzi et al., 2009).

Further studies have instead suggested that the amygdala processes affective valence (Murray, 2007). Although many neuroimaging and lesion studies have shown that the amygdala is more responsive to negative than positive affective stimuli (Adolphs et al., 1994; Hamann et al., 1996; Morris et al., 1996; Costafreda et al., 2008), two large meta-analyses revealed a greater effect size for positive compared with negatively valenced cues (Sergerie et al., 2008; Fusar-Poli et al., 2009). Of the five maternal response studies that also explored affect valence (i.e., happy and sad infant faces) (Noriuchi et al., 2008; Strathearn et al., 2008; Lenzi et al., 2009; Strathearn et al., 2009; Barrett et al., 2011), only one reported a significant main effect of valence on amygdala activation, and only when contrasting combined affect groups (happy/sad/ambiguous faces) with neutral faces (Lenzi et al., 2009). In our own previous work, we specifically contrasted affectively valenced cues (happy and sad infant faces) with neutral face cues, but found no significant amygdala activation in first-time mothers (Strathearn et al., 2008, 2009).

Still other studies have proposed that the amygdala responds to generalized arousal, or stimulus intensity, regardless of whether the valence is positive or negative (Anderson et al., 2003; Small et al., 2003; Winston et al., 2005). However, this concept has also been questioned by studies demonstrating amygdala activation independent of arousal (Ewbank et al., 2009; Vrticka et al., 2012).

In attempting to synthesize all of these findings on amygdala response with regard to interpersonal cues, affective valence, and arousal, a growing body of literature has suggested that the amygdala may be best characterized as a center for appraising absolute “value”—or biological relevance—of affective stimuli (Sander et al., 2003; Belova et al., 2008; Morrison and Salzman, 2010; Vrticka et al., 2012). Thus, others have proposed that the amygdala may function as a “relevance detector,” integrating these input signals with decision-making and reward processing regions of the brain in order to determine the likelihood of approach or withdrawal behavior (Murray, 2007; Morrison and Salzman, 2010; Ousdal et al., 2012).

Vrticka et al. (2012) recently studied amygdala response to “social relevance” in women, comparing responses to social vs. non-social scenes, while contrasting affective valence and controlling for differences in arousal. The authors identified a significant interaction effect between social content and affect valence (positive vs. negative), which was also seen in other cortical regions. This suggested that the amygdala might be part of a distributed cortical and sub-cortical network for relevance detection.

In the present study of first-time mothers, we examined the role of the amygdala in processing socially relevant positive and negative infant face cues, adding the dimension of “personal relevance” by comparing responses to own-infant vs. unknown-infant faces. Firstly, in view of previous whole-brain analyses showing no main effect of valence on amygdala response in mothers (Noriuchi et al., 2008; Strathearn et al., 2008, 2009; Barrett et al., 2011), we tested whether this effect would emerge at the level of an anatomically defined amygdala region of interest (ROI). We compared the presence or absence of affect by contrasting happy or sad with neutral infant faces. Next, we explored whether, in the presence of positive or negative face affect, there was an interaction effect with infant identity. Using a sample of mothers almost twice the number of any previous maternal brain study, we also adjusted for self-reported arousal. Finally, we looked for similar effects in other cortical and subcortical regions, as part of a whole-brain analysis.

We hypothesized that a mother's amygdala response to happy or sad infant face cues would be moderated by personal relevance, independent of arousal, and would be associated with activation of other brain regions related to maternal caregiving behavior.

## Materials and methods

### Participants

Thirty-nine first-time mothers (age: 28.5 ± 0.8 years; 74% married; 64% Caucasian, 13% African American, 18% Hispanic, and 5% Other; Full Scale IQ estimate: 109.5 ± 1.3) participated in the present study. Participants were recruited as part of a larger study through community advertisements and local prenatal clinics. All participants were right-handed, were free of nicotine use during pregnancy, and were not on psychotropic medications at the time of study enrollment. At the time of the scanning visit, only two of the mothers screened positive for mild symptoms of depression, based on the Beck Depression Inventory-II (Beck et al., 1996). There were no self-reports of current or past alcohol or drug abuse problems or involvement in substance abuse treatment programs. Each participant provided written informed consent in accordance with the protocol approved by the institutional review board at Baylor College of Medicine.

### Study design

Sixty-one participants met study criteria and were recruited during the third trimester of pregnancy. Approximately 7 months after delivery, enrolled women and their infants attended a video-recording session during which smiling, crying and neutral face images were collected from each infant (age of infant: 6.8 ± 0.3 months) and prepared for use in the subsequent scanning session. Approximately 11 months after delivery, 44 mothers underwent fMRI scanning while passively viewing face images of both their own infant and a single matched unknown infant. Upon completion of the scan, 39 mothers completed ratings of their level of emotional arousal (0 = calm and 8 = aroused) for each of the infant-face images shown in the scanner, using a 9-point scale adapted from the Self-Assessment Manikin (Bradley and Lang, 1994). In addition, they rated valence of the face images (0 = positive, 4 = neutral, 8 = negative), both from their own perspective and the perspective of the infant, responding to the questions: “How pleasant or unpleasant did the picture make you feel?” and “How do you think the baby was feeling?” (hereafter referred to as “mother's feelings” and “mother's perception of infant feelings,” respectively). There was a minimum interval of 3 months between the videotaping and scanning visits, with a mean interval of 4.4 ± 0.5 months.

### Stimuli

Experimental stimuli consisted of 60 infant-face images, 30 of the mother's own infant and 30 of the matched unknown infant. The still face images were captured from a video recording and sorted into one of three affect valence groups: happy, neutral, or sad. Each infant was then matched with a single control infant, unknown to each mother, with an equal number of images from each affect group. The two infants were also matched on age and race (and sex if distinguishable). The “own-infant” faces for one mother, were also used as “unknown-infant” faces for another mother whenever possible, although we were not able to perform pair-wise matching for all mothers because of variation in infant age and race. Final stimuli consisted of six face categories, own-happy (OH), own-neutral (ON), own-sad (OS), unknown-happy (UH), unknown-neutral (UN), and unknown-sad (US), each containing 10 unique images. Three independent female raters confirmed that own and unknown infant images did not differ significantly in terms of positive and negative valence [*t*_(38)_ = −1.31, *p* = 0.20 for OH vs. UH; *t*_(38)_ = −0.32, *p* = 0.75 for OS vs. US] or infant gaze direction (direct or averted gaze; own vs. unknown; all *p*s > 0.60). The images were projected onto an overhead mirror display for viewing during fMRI scanning. All 60 images were presented in a pseudorandom order as part of an event-related design in a single fMRI run, and were repeated in a second run. Images were not repeated within each run. The stimulus duration was 2 s and the inter-stimulus interval randomly varied between 2, 4, and 6 s.

### Functional MRI data acquisition and preprocessing

Imaging was performed on a 3-Tesla Siemens Allegra scanner. High-resolution T1-weighted anatomical images were acquired (192 slices; in plane resolution, 256 × 256; field of view, 245 mm; slice thickness, 1 mm), followed by two whole-brain blood oxygenation level-dependent (BOLD) functional runs of about 185 scans each, using a gradient recalled echo planar imaging sequence (37 slices; repetition time, 2000 ms; echo time, 25 ms; flip angle, 90°; matrix, 64 × 64; field of view, 220 mm; slice thickness, 3 mm). Axial slices were positioned at 30° to the line connecting the anterior and posterior commissures. The first and second functional runs are hereafter referred to as early and late phases, respectively.

Imaging data for each subject was preprocessed using the BrainVoyager QX software (Version 1.7.9, Brain Innovation, Maastricht, The Netherlands; Goebel, 2006). Images were corrected for slice timing and realigned to the first volume for head motion correction. Functional data were then coregistered with the anatomical data, transformed into 3 × 3 × 3 mm isotropic voxels, and then normalized into the Talairach space. Further details of preprocessing can be found in Strathearn et al. (2008).

### Statistical analysis

#### Behavioral data analysis

Mothers' rating data were inspected for normality, and mothers' emotional arousal ratings were log-transformed to optimize the approximation to normal distribution. Mothers' valence (i.e., mother's feelings and mother's perception of infant feelings) and arousal ratings were separately examined in repeated-measures ANOVAs, with infant affect valence (happy, sad and neutral) and identity (own vs. unknown) as within-subject factors. The association between mothers' self-reported arousal and amygdala BOLD response was examined in a correlation analysis.

#### Functional MRI data analysis

**Figure 1:**
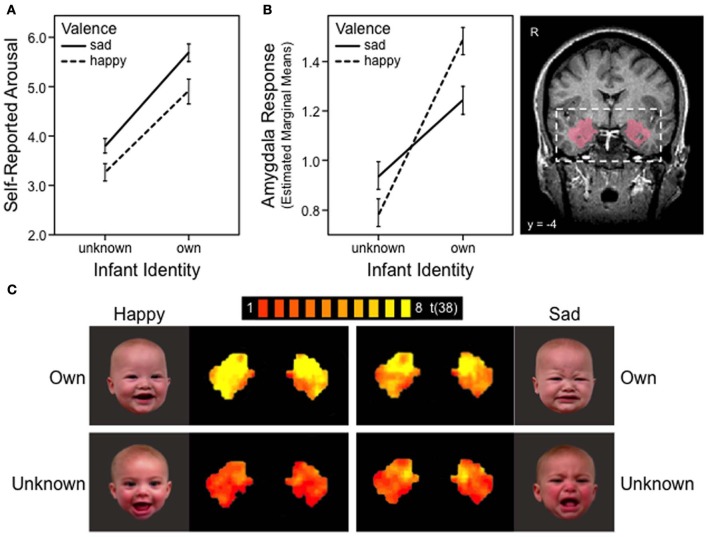
**Maternal responses to own and unknown infant face cues, happy vs. sad. (A)** Mother's self-reported emotional arousal, rated using a 9-point Likert scale: 0 = calm and 8 = aroused. Error bars depict standard error of mean. **(B)** BOLD response in the bilateral amygdala region of interest. Anatomical mask used to define amygdala (probabilistic map) is shown on the right. Error bars depict standard error of mean. **(C)** FMRI activation map of the bilateral amygdala in response to four infant face categories: own happy, own sad, unknown happy and unknown sad. Maps presented with false discovery rate corrected threshold, *q* < 0.05.

A general linear model (GLM) was specified for each subject, and each predictor (i.e., OH, OS, ON, UH, US, and UN) was convolved with a double-gamma hemodynamic response function. The resulting reference time courses were used to model the signal time course at each voxel and to calculate parameter estimates (β) for each predictor. These individual estimates were submitted to a second-level random-effects analysis within the anatomically defined ROI, bilateral amygdala. The mask was obtained from the SPM Anatomy Toolbox (Eickhoff et al., 2005) and was based on the probabilistic location of basolateral amygdala in adult humans, taking into account intersubject neuroanatomical variability (Amunts et al., 2005), transformed into Talairach space. It consisted of 319 contiguous voxels on each side of the brain (Figure 1B).

To confirm the previous whole-brain results (Noriuchi et al., 2008; Strathearn et al., 2008, 2009; Barrett et al., 2011) at the level of ROI analyses, we first probed for a significant main effect of valence within the amygdala ROI. The *z*-normalized BOLD signals were extracted from the bilateral amygdala mask, and within-subject differences between affect conditions (i.e., happy, sad, neutral) were examined via repeated measures ANOVAs and *post-hoc* comparisons of means.

The BOLD data were then submitted to mixed-effects linear regression analysis to examine how infant identity may interact with affective valence (positive vs. negative) to modulate mothers' amygdala response. The mixed-effects models were built as follows: (a) the initial model included the fixed main effects of identity (own vs. unknown), valence (happy vs. sad), and laterality (left vs. right amygdala). Phase (early vs. late) was initially included in the model to examine habituation between phases; (b) subject-level random intercept and slope were added to model systematic inter-individual variability; (c) interaction terms were added sequentially and retained in the model if they improved model fit; (d) mothers' self-reported emotional arousal was added as a covariate to examine whether variability in mothers' emotional arousal altered the significance of the model fit and parameter estimates. The best-fit model was identified using maximum likelihood estimation, and likelihood-ratio chi-square tests were used to assess the relative fit of nested models.

The optimal model [Wald χ^2^(4) = 37.24, *p* < 0.0001] consisted of a random effects structure that included a subject-level random intercept [LR χ^2^(1) = 99.74, *p* < 0.0001] and a random slope for identity [LR χ^2^(2) = 28.27, *p* < 0.0001]. SPSS version 21 and STATA/SE, version 12.1 (STATA Corp, College Station, TX) were used in all ROI analyses.

The ROI analyses were followed by whole-brain analyses to evaluate the context of the ROI findings. The hypothesized within-subject interaction between identity and affective valence was examined in an identity (own vs. unknown) × valence (happy vs. sad) random-effects ANOVA and specific identity and valence contrasts were examined. A cluster threshold of ≥ 100 mm^3^ was used to determine clusters of significant activation.

## Results

### Behavioral rating data

Means and standard deviations of the mothers' ratings are shown in Table 1 for the six categories of infant faces.

**Table 1:** **Mothers' self-reported ratings (*M* ± SD) of infant face stimuli (own and unknown)**.

**Valence**	**Mother's feelings^a^**		**Mother's perception of infant feelings^b^**		**Emotional arousal rating^c^**	
	**Own**	**Unknown**	**Own**	**Unknown**	**Own**	**Unknown**
Happy face	1.15 ± 0.76	2.71 ± 0.84	1.21 ± 0.78	1.55 ± 0.83	4.91 ± 2.24	3.26 ± 1.58
Neutral face	2.81 ± 0.85	3.86 ± 0.47	3.60 ± 0.59	3.93 ± 0.65	4.01 ± 1.60	2.67 ± 1.32
Sad face	6.25 ± 1.18	5.23 ± 0.83	6.91 ± 0.70	6.80 ± 0.81	5.69 ± 1.66	3.80 ± 1.36

The ratings using 9-point Likert scales adapted from the Self-Assessment Manikin (Bradley and Lang, 1994) with the following benchmarks: 0 = positive, 4 = neutral, 8 = negative for mother's feelings and mother's perception of infant's feelings ratings; 0 = calm, 8 = aroused for emotional arousal rating.

a “How pleasant or unpleasant did the picture make you feel?” Main effect of valence: F_(2, 76)_ = 281.28, p < 0.001.

b “How do you think the baby was feeling?” Main effect of valence: F_(2, 76)_ = 713.83, p < 0.001.

c While statistical tests were conducted using log-transformed data, untransformed data are reported here for clarity of interpretation.

Main effect of valence: F_(2, 76)_ = 20.73, p < 0.001; Main effect of identity: F_(1, 38)_ = 42.98, p < 0.001. No interaction effect.

#### Affect valence

Mothers' ratings confirmed that happy, neutral, and sad faces were significantly different in terms of perceived valence. For both own and unknown infants, happy faces were rated as significantly more positive (i.e., pleasant), while sad faces were rated as significantly more negative (i.e., unpleasant), compared to neutral faces (all *p*s < 0.001).

#### Arousal

For mothers' self-reported arousal, significant main effects were found for both identity [*F*_(1, 38)_ = 42.98, *p* < 0.001] and affect valence [*F*_(2, 76)_ = 20.73, *p* < 0.001], with no significant interaction between the two [*F*_(2, 76)_ = 0.34, *p* = 0.71]. Across all three affect groups, mothers reported greater emotional arousal when viewing their own infant's face compared to the unknown infant's face (all *p*s < 0.001). Regardless of infant identity, sad infant faces elicited the greatest emotional arousal, followed by happy faces, with neutral faces showing the least level of arousal (all *p*s < 0.05) (Table 1). Mothers' self-reported arousal ratings were significantly and positively correlated with their bilateral amygdala BOLD response (*r* = 0.29, *p* < 0.001).

### Neuroimaging data

Consistent with previous research documenting amygdala habituation over time (Breiter et al., 1996), we found evidence of habituation in the late phase (i.e., run 2). Analyses of both phases, with phase as a within-subject factor, yielded results largely similar to those described below (i.e., results obtained when examining early phase only). However, in this analysis, significant main and interaction effects were modified by their interactions with phase, revealing that the effects were significantly reduced in the late phase compared to the early phase. In fact, all effects were reduced to non-significance when examining the late phase data only. Given the evidence of habituation, we focus below on the results from the early phase data.

#### Affect valence

Means and standard errors of amygdala BOLD responses are presented in Table 2 for the six stimulus categories.

**Table 2:** **Mothers' amygdala BOLD responses to infant face stimuli**.

**Valence**	**Infant Identity**	
	**Own**	**Unknown**
Happy face	1.84 ± 0.15	0.96 ± 0.15
Neutral face	1.73 ± 0.18	1.11 ± 0.13
Sad face	1.58 ± 0.15	1.16 ± 0.15

Values (M ± SE) represent z-normalized BOLD signal change values extracted from the anatomically defined bilateral amygdala mask. There was no effect of laterality; data from the right and left amygdala are hence collapsed.

Firstly, we tested whether the amygdala response was moderated by the presence or absence of infant face affect, comparing happy and sad with neutral faces. We confirmed that there was no main effect of valence when using an ROI analysis of the amygdala [*F*_(2, 76)_ = 1.44, *p* = 0.24 for own; *F*_(2, 76)_ = 1.05, *p* = 0.36 for unknown]. Specifically, no significant differences were found between mothers' amygdala response to happy vs. neutral [*t*_(38)_ = 0.77, *p* = 0.45 for own; *t*_(38)_ = −1.08, *p* = 0.29 for unknown], sad vs. neutral [*t*_(38)_ = −0.95, *p* = 0.35 for own; *t*_(38)_ = 0.28, *p* = 0.78 for unknown], or affective (i.e., happy and sad combined) vs. neutral faces [*t*_(38)_ = −0.14, *p* = 0.89 for own; *t*_(38)_ = −0.38, *p* = 0.70 for unknown], for either own or unknown infant faces. Thus, in first-time mothers, the amygdala did not respond specifically to the presence of affect in infant face cues, comparing happy or sad affect with neutral.

Next, we examined whether, in the presence of affect, the amygdala response was modulated by the valence of affective cues present (i.e., positive vs. negative directionality). We confirmed that there was no main effect of valence. No significant difference was found between mothers' amygdala response to happy vs. sad [*t*_(38)_ = 1.64, *p* = 0.11 for own; *t*_(38)_ = −1.62, *p* = 0.11 for unknown].

#### Identity and valence x identity interaction

We then tested whether the amygdala response to affectively valenced cues (positive or negative) would be moderated by infant identity. Results are illustrated in Figure 1; the figure also presents results of the self-reported arousal for comparison. We found a significant main effect of identity (β = 0.35, 95% CI = 0.11–0.59, *z* = 2.87, *p* = 0.004; Figure 1B), consistent with findings from the self-reported arousal ratings (Figure 1A). However, unlike self-reported arousal ratings, the effect of identity in amygdala response was qualified by a significant identity x valence interaction effect (β = 0.39, 95% CI = 0.15–0.62, *z* = 3.24, *p* = 0.001; Figures 1B,C). Decomposition of the interaction revealed that mothers' amygdala response was significantly greater for happy than sad faces of their own infant (coefficient = 0.23, *z* = 2.67, *p* = 0.008), whereas the reverse pattern was observed for unknown infant faces, with marginal significance (coefficient = −0.16, *z* = 1.91, *p* = 0.056). The amygdala response for own-infants was significantly greater than that of unknown-infants, for both happy (coefficient = 0.74, *z* = 6.06, *p* < 0.001) and sad (coefficient = 0.35, *z* = 2.87, *p* = 0.004) faces (Figures 1B,C). No differences were found between the left and right amygdala (β = 0.02, 95% CI = −0.10–0.14, *z* = 0.34, *p* = 0.737).

When self-reported emotional arousal was added to the model, it did not significantly predict amygdala response, above and beyond that which was predicted by infant identity and valence (β = −0.20, 95% CI = −0.70–0.30, *z* = −0.78, *p* = 0.433). In fact, the model fit and significant results were essentially unchanged when arousal was added to the model [Wald χ^2^(5) = 37.96, *p* < 0.0001].

#### Whole brain analysis

**Figure 2:**
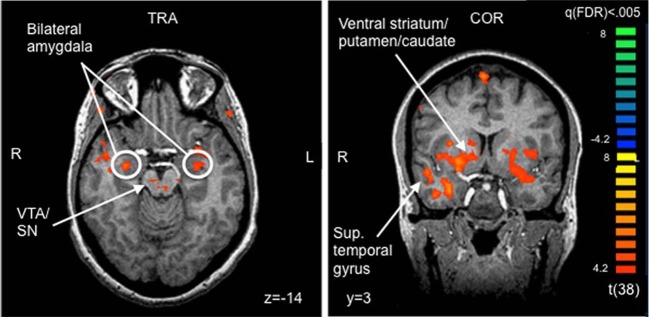
**Selected areas of significant activation from Own Happy > Unknown Happy contrast.** FDR corrected *q* < 0.005, cluster threshold ≥ 300 mm^3^. VTA, ventral tegmental area; SN, substantia nigra; TRA, transverse slice; COR, coronal slice; FDR, false discovery rate.

On whole-brain analysis, the identity (own vs. unknown) × valence (happy vs. sad) ANOVA yielded no significant findings at a statistical threshold of FDR corrected *q* < 0.05. However, an identity × valence interaction effect was seen in the amygdala at the less stringent threshold of *p* < 0.005 (uncorrected), confirming the ROI finding. The identity × valence interaction effect also emerged in several additional regions that were not of *a priori* interest, including the prefrontal cortex, superior and middle temporal gyri, and the thalamus (Table 3). Activation was also seen in the amygdala for the OH vs. UH contrast, but not for OS vs. US (all at FDR corrected, *q* < 0.005), similar to the reported findings in Strathearn et al. (2008) (Figure 2). The OH vs. UH contrast also yielded significant activation in dopamine-related reward processing regions (ventral tegmental area/substantia nigra region, ventral and dorsal striatum), and the superior temporal gyrus, an area involved in emotion and face processing, overlapping previously reported activation patterns from a subset of this study sample (Strathearn et al., 2008) (Table 4; Figure 2).

**Table 3:** **Areas of infant identity x affect interaction in whole brain analysis**.

	**Hemisphere**	**Talairach coordinates**			**Volume (mm^3^)**	**Peak *F* value**	***p***
		***x***	***y***	***z***			
**FRONTAL LOBE**							
Medial frontal gyrus (BA 10)	Left	−7	64	9	1072	39.42	<0.00001
Precentral gyrus (BA 4)	Right	47	−11	48	111	16.35	0.00025
Superior frontal gyrus (BA 6)	Left	−10	−5	69	338	15.30	0.00037
**PARIETAL LOBE**							
Postcentral gyrus (BA 2/3)	Left	−52	−20	36	676	22.21	0.00003
**TEMPORAL LOBE**							
Superior temporal gyrus (BA 22)	Right	35	−53	12	124	23.32	0.00002
Middle temporal gyrus (BA 21/38)	Right	41	7	−33	230	19.89	0.00007
**LIMBIC LOBE / SUB-LOBAR REGIONS**							
Thalamus	Right	11	−20	6	285	22.48	0.00003
Amygdala / Claustrum	Left	−31	1	−9	115	17.12	0.00019
**CEREBELLUM**							
Cerebellum / (Fusiform gyrus)	Left	−31	−47	−27	282	21.34	0.00004
Culmen	Right	17	−26	−30	176	18.24	0.00013

p < 0.005 (uncorrected), cluster threshold ≥ 100 mm^3^; Talairach coordinates (x, y, z) represent peak voxels in each cluster; BA, Brodmann's area.

**Table 4:** **Areas of significant activation from Own Happy > Unknown Happy contrast in whole brain analyses**.

	**Hemisphere**	**Talairach coordinates**			**Volume (mm^3^)**	**Peak *t* value**	***p***
		***x***	***y***	***z***			
**FRONTAL LOBE**							
Precentral gyrus (BA 4)	Right	53	−8	42	1909	8.88	<0.000001
Superior frontal gyrus (BA 6)	Right	2	4	66	805	6.73	<0.000001
Inferior frontal gyrus (BA 13)	Left	−40	22	12	405	7.43	<0.000001
**PARIETAL LOBE**							
Postcentral gyrus (BA 3)	Left	−52	−17	36	1515	6.49	<0.000001
**TEMPORAL LOBE**							
Superior temporal gyrus (BA 38)	Right	35	10	−24	2043	7.16	<0.000001
Superior temporal gyrus (BA 38)	Right	44	4	−15	398	5.62	0.000002
**LIMBIC LOBE / SUB-LOBAR REGIONS**							
Ventral striatum / Putamen	Right	23	−17	6	3515	7.48	<0.000001
Amygdala / Dorsal striatum / Claustrum	Left	−31	−2	−6	6120	7.28	<0.000001
Dorsal Caudate	Right	11	10	9	349	6.50	<0.000001
Dorsal Putamen	Right	29	−8	9	480	5.82	0.000001
**MIDBRAIN**							
Substantia nigra / VTA region	Right	14	−20	−6	763	6.35	<0.000001
Substantia nigra / VTA region	Left	−4	−29	−30	339	5.60	0.000002
**CEREBELLUM**							
Culmen	Right	20	−29	−30	417	6.96	<0.000001

FDR corrected q < 0.005, cluster threshold ≥ 300 mm^3^; Talairach coordinates (x, y, z) represent peak voxels in each cluster; BA, Brodmann's area; VTA, ventral tegmental area.

## Discussion

The relationship between a mother and her infant is a uniquely personal experience, forged through nine months of prenatal interaction and communication, dramatic hormonal changes accompanying pregnancy and childbirth, and direct somatosensory exchanges that occur during feeding and lactation (Levy et al., 2011). Understanding, prioritizing and responding to infant cues is an important capacity of motherhood, with specific brain mechanisms evolving to facilitate this need (Kinsley et al., 1999; Kinsley and Amory-Meyer, 2011).

The present study examined how the amygdala, and associated brain networks, assist mothers to respond most adaptively to infant face cues. Rather than showing an affect-specific response for either happy or sad faces, as has been traditionally understood (Murray, 2007), we found that a mother's amygdala response was modulated by the identity of the infant face. Both amygdala activation and corresponding arousal ratings were greater when mothers viewed their own infant's face compared to unknown infant faces, regardless of infant affect valence (Figure 1). Likewise, sad faces of unknown infants produced greater emotional arousal than happy faces, and tended to elicit greater amygdala activation. However, the inverse was true when mothers viewed their *own* infants' faces: amygdala activation was greater for happy compared to sad faces, despite less self-reported emotional arousal for happy faces. Our study also found that self-reported arousal did not predict amygdala response after accounting for these other aspects—face identity and affect valence, confirming that the amygdala does not solely represent an arousal response in the brain (Ewbank et al., 2009; Vrticka et al., 2012).

These results are consistent with the view that the amygdala functions as a “relevance detector,” a concept first proposed by Sander et al. (2003). “Relevance,” as a psychological concept derived from appraisal theory of emotion, stresses “the contextual and goal-dependent value of a stimulus within a *personal* situation” (Adolphs, 2010). A recent fMRI study confirmed that the amygdala responds preferentially to highly relevant cues, compared to less relevant cues, with functional connectivity seen between the amygdala and the ventral striatum, a key reward processing region (Ousdal et al., 2012). On contrasting own vs. unknown happy faces, we likewise saw activation of both the amygdala and the ventral striatum and other dopamine-associated reward areas of the brain. For mothers responding to infant affective cues, assessing “relevance” involves weighing the connectedness of a relationship as well as the significance of the affective valence cues.

With unknown or unfamiliar face cues, like those used in almost all prior studies of the amygdala (Sergerie et al., 2008), negative stimuli may be more salient to the individual in order to elicit a self-protective or withdrawal response. Our results, in this respect, were consistent with a study of mothers responding to infant cries vs. laughter, which showed that cries from an unknown infant produced greater amygdala activation than laughter (Seifritz et al., 2003). No other study has contrasted a mother's own infant cry vs. laughter, although one study of own vs. unknown infant cry also revealed greater amygdala activation, as we have shown for face affect, but in breastfeeding vs. bottle-feeding mothers (Kim et al., 2011).

When a personally relevant cue is presented, such as when a mother views her own infant's face, positive cues may result in a higher value computation in the amygdala, compared with negative cues (e.g., Lenzi et al., 2009). In non-attachment contexts, negative cues may be more relevant in mobilizing a response (either withdrawal for self-protection, or an altruistic helping response). However, in attachment contexts, smiling infant faces may be more salient, as they form the basis of the attachment approach system, and activate reward processing brain regions, such as the striatum and medial prefrontal cortex, as noted in both this study and previously published reports (Strathearn et al., 2008, 2009). Nevertheless, own-infant sad faces still elicit a stronger amygdala response than either happy or sad *unknown* faces, suggesting that own-sad cues are still a highly relevant signal.

Several other studies of maternal brain response to infant face cues have shown a difference in amygdala activation based on infant identity (Bartels and Zeki, 2004; Leibenluft et al., 2004; Ranote et al., 2004; Barrett et al., 2011). However, only one of these studies explored differences related to infant affect valence. In the study by Barrett et al. (2011), differences in amygdala activation were seen for own vs. unknown infant faces, but only in positive and not negative faces. A significant interaction effect was not reported. Own-infant positive faces also activated the amygdala more than negative faces, although the difference was not statistically significant, and no difference was seen for unknown-infant faces. Having almost twice the number of mothers participating in the current study enabled us to use more sophisticated analysis techniques on an anatomically defined amygdala ROI, which demonstrated our highly significant interaction effect.

Oxytocin is a neuropeptide with a localized effect within the amygdala (Kirsch et al., 2005; Baumgartner et al., 2008; Petrovic et al., 2008; Domes et al., 2010). It is produced in response to personally relevant social cues (Feldman, 2012)—such as mothers interacting with their own infants (Strathearn et al., 2009). In fact, central oxytocin facilitates the onset of offspring-specific maternal behavior in sheep, in which ewes lick and suckles their own lamb, while avoiding or aggressively rejecting any other approaching lambs (Keverne and Kendrick, 1992). One fMRI study of intranasal oxytocin using unknown face cues, revealed greater amygdala activation to *negative* faces in placebo condition, but greater activation to *positive*, smiling faces after intranasal oxytocin (Gamer et al., 2010). It is intriguing to postulate whether endogenous oxytocin, produced in response to personally relevant infant cues (Strathearn et al., 2009), may be driving the personal relevance effects seen in the present study.

Although we have argued that the observed amygdala responses to own and unknown infant face cues are indicative of “personal relevance,” other unmeasured factors may also be involved, such as motivational state or other psychological traits (Canli et al., 2001; Vrticka et al., 2008, 2012). However, the idea that amygdala activation in mothers is an indication of “vigilant protectiveness” (Leibenluft et al., 2004; Gobbini and Haxby, 2007) seems less likely, in view of our finding of heightened response to smiling vs. crying own-infant faces. Although novelty has also been associated with amygdala response (Blackford et al., 2010; Weierich et al., 2010; Balderston et al., 2011), in our study the more novel unknown faces did not produce an increased amygdala response.

Although we talk about the amygdala as a single entity, it is actually composed of a diverse number of nuclei and cell types (Murray, 2007), with individual neurons that respond to particular stimulus categories, such as emotional valence or face identity (Gothard et al., 2007). Amygdala neurons may develop functional specificity in response to repeated exposure to affective stimuli during development (Tottenham, 2012). Chronic exposure to danger or stress, such as occurs with child maltreatment, is associated with hyper-reactivity of the amygdala in response to negative (but not positive) unknown faces (Dannlowski et al., 2012a). In contrast, post-natal depressive and anxiety symptoms [which may also be associated with childhood maltreatment (Grant et al., 2011; McCrory et al., 2011)] are related to a diminished amygdala response to faces (Moses-Kolko et al., 2010; Barrett et al., 2011). Thus, one's perception of relevance and amygdala response may depend not only on present affective cues, but also on prior experience.

Understanding how the amygdala processes affective information and detects personal relevance in infant cues may help us to better understand its role in a host of psychiatric disorders affecting motherhood, including post-partum depression (Moses-Kolko et al., 2010), post-traumatic stress disorder (Bremner, 2003; Dannlowski et al., 2012b) and maternal addiction (Landi et al., 2011). This study demonstrates that positive facial expressions from one's own infant may be an important area of focus.

### Conflict of interest statement

The authors declare that the research was conducted in the absence of any commercial or financial relationships that could be construed as a potential conflict of interest.
